# Extraction of Protein-Protein Interaction from Scientific Articles by Predicting Dominant Keywords

**DOI:** 10.1155/2015/928531

**Published:** 2015-12-10

**Authors:** Shun Koyabu, Thi Thanh Thuy Phan, Takenao Ohkawa

**Affiliations:** Graduate School of System Informatics, Kobe University, 1-1, Rokkodai, Nada, Kobe 657-8501, Japan

## Abstract

For the automatic extraction of protein-protein interaction information from scientific articles, a machine learning approach is useful. The classifier is generated from training data represented using several features to decide whether a protein pair in each sentence has an interaction. Such a specific keyword that is directly related to interaction as “bind” or “interact” plays an important role for training classifiers. We call it a dominant keyword that affects the capability of the classifier. Although it is important to identify the dominant keywords, whether a keyword is dominant depends on the context in which it occurs. Therefore, we propose a method for predicting whether a keyword is dominant for each instance. In this method, a keyword that derives imbalanced classification results is tentatively assumed to be a dominant keyword initially. Then the classifiers are separately trained from the instance with and without the assumed dominant keywords. The validity of the assumed dominant keyword is evaluated based on the classification results of the generated classifiers. The assumption is updated by the evaluation result. Repeating this process increases the prediction accuracy of the dominant keyword. Our experimental results using five corpora show the effectiveness of our proposed method with dominant keyword prediction.

## 1. Introduction

Proteins and their interactions play a leading role in the most fundamental biological processes, including metabolic activity, signal transduction, and DNA replication and transcription. In general, proteins express their functions through interaction with other molecules, including other proteins.

The information of a protein-protein interaction (PPI) can be found in the scientific literature. Although many efforts have created databases that store PPIs in computer readable form as structured data, it still takes too much time and labor to extract these valuable sources from the scientific literature. As a result, in recent years, much research has addressed the automated extraction of PPI information from biological literature.

For the automatic extraction of PPI, the machine learning technique is often utilized. In such approaches, classifiers are created to identify whether there is an interaction between two proteins appearing in a sentence. Many methods that apply the machine learning technique have been proposed, and it is very common to adopt supervised learning, which uses explicit PPI information as training data.

In most of these methods, a protein pair, which consists of two protein names appearing in a sentence, is regarded as an instance along with this sentence. Each instance is represented by many features including lexical features, word context features, and syntactic features derived from the sentence or its syntactic structure. A classifier is trained from instances given as a set of feature vectors. For example, the method proposed by Bunescu and Mooney learned extraction patterns for PPI with a generalized subsequence kernel that utilizes the following three patterns in a sentence: before the first protein, between two proteins, and after the second protein [1]. Besides global context kernels, Giuliano et al. also exploited a local context kernel around the interacting entities and a shallow linguistic kernel [2]. Mitsumori et al. trained the word features in a sentence around the protein names [3].

In such frameworks, certain feature values in some instances, which could determine very clearly whether there are interactions in them significantly impact the training of the classifiers. The presence of such keywords related to interaction as “*interact*” and “*bind*” is a typical example of such feature values. We call keywords with prominent contributions when creating classifiers* dominant keywords*.

Dominant keywords are exceptionally effective in the process of training classifiers. However, note that the existence of some dominant keywords renders other features ineffective. In other words, dominant keywords might become double-edged swords and decrease the accuracy of training classifiers. Therefore, when dominant keywords greatly contribute to training classifiers, more focus should be exerted on the keyword features. In the opposite case, on the contrary, it is important to give significant consideration to other features. Moreover, not only dominant keywords but also some features play important roles depending on the sentence structure.

In this paper, we propose a novel method in which a training set is divided into four subsets based on the dominant keywords and the sentence structure (the position of the keyword) in each instance and four types of classifiers are generated from each subset to improve the classification accuracy. If the training set covers all of the possible instances completely, a keyword, which can determine whether the instance with this keyword belongs to a positive class (including PPI) or a negative class (excluding PPI), can be considered dominant. However, the training set contains unbalanced data biased toward negative instances including no PPI and is created manually. Therefore, it is very difficult to determine if the bias in the classification into classes is due to these keywords or that the instances using these keywords are gathered in only one class by chance. Furthermore, whether a keyword is* dominant* is not absolute. In several instances, a keyword plays a leading role in classification as a dominant keyword; conversely, in other instances, the same keyword might not fulfill its role as a dominant keyword. For example, in some typical sentence patterns, the keyword “interact” is probably important evidence in identifying the presence of PPI. But, in other sentence patterns, other features may become the main factors rather than the keyword “interact” in determining classification. With respect to the former examples, “interact” is considered a dominant keyword, but, with respect to the latter, it is* not* considered a dominant keyword.

Therefore, we introduce a mechanism that can predict whether the mentioned keyword is dominant for each instance. Initially, we assume that a keyword is a dominant keyword based on the bias of the classes in the instance that contains it. After the training set is divided into two subsets, one consisting of instances with one of the initially assumed dominant keywords and another consisting of the instances without the assumed dominant keywords, two classifiers are generated by training these two subsets. Based on the classification result of these two classifiers, we verify the reasonableness of the presence or the absence of the dominant keyword that was assumed previously and update this assumption. By repeating these verification and assumption processes of the dominant keyword, we can obtain a more appropriate division of subsets. On the other hand, with respect to the division of subsets based upon differences in the sentence structure, we do not use the verification and assumption processes shown in the prediction of dominant keywords. Instead, we exploit the sentence patterns provided beforehand. Since several features are useless for particular sentence patterns, they are removed by feature selection to improve the extraction accuracy.

The rest of this paper is organized as follows. In the next section, we show features that represent the instances in our proposed method. In Section 3, we describe its details. First, dominant keywords are introduced. Then we describe the schema of PPI extraction by dividing the training set into subsets based on the dominant keywords and the position of keywords. Predictions about the presence of dominant keywords in every instance and the method of feature selection concerning sentence structure are also mentioned. In Section 4, we describe our experiments that evaluated the effectiveness of our extraction method for PPI information and compare it with other methods. Finally, we present a final conclusion and discuss future challenges.

## 2. Features of Protein Pairs in Sentences

We consider a binary classification problem in which we deal with positive instances that include PPI and negative instances that do not include it. In the automatic PPI extraction approach, criteria for distinguishing between positive and negative instances, which are identified beforehand, are automatically found using the characteristics of the sentences containing the protein pairs. In this paper, we refer to these sentence characteristics as features.

The PPI extraction framework is described as follows:

- The training data are given by a list of features (feature vectors) and their known class labels.
- Based on the training data, we perform the machine learning algorithm and train the classifiers.
- The test data whose feature vectors are known beforehand (but no class label) are given to the classifiers. The classifiers output the prediction results that identify whether a PPI exists between any protein pair.

The features obtained from a sentence related to PPI may be a description that directly expresses PPI, the existence of words implying PPI, or a description that shows that no PPI exists. These features have been used in many studies of PPI information extraction [3–6]. In this paper, we set several features by extending these features.

The features used in this paper are broadly divided into three categories: features obtained directly from the sentence, those obtained from parsing information, and those using existing patterns. Next we describe them in detail. In the following tables, *P*1, *P*2, and *K* denote the protein name appearing first, that appearing later, and the keyword, respectively. The value of each feature is determined for a pair of protein names.

### 2.1. Features Obtained Directly from Sentences

Features that can be extracted from sentences in the text are summarized in Table 1.

### 2.2. Features Obtained from Parsing Information

**Figure 1:**
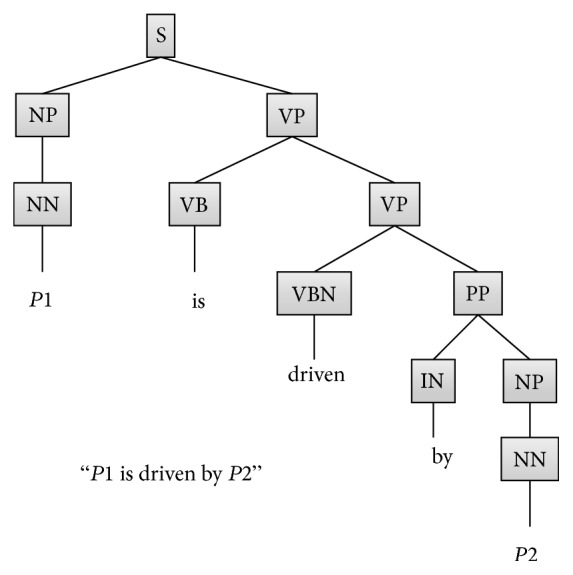
Example of parse tree.

The syntactic structure of the sentence is expressed by parse trees. From them, we can clarify such syntactic features of sentences as the structure of phrases and the structural relation of word pairs. The features obtained from a parse tree are shown in Table 2. An example of a parse tree generated by the Stanford parser (http://nlp.stanford.edu/software/lex-parser.shtml) is shown in Figure 1.

### 2.3. Features Using Existing Patterns

Several structure patterns are related to the presence or absence of PPI [7]. Based on the structure patterns [8], we prepared thirteen kinds of structure patterns in Table 3. If a protein pair (*P*1, *P*2) matches (or does not match) one of these patterns, we use “true” (or “false”) as a feature value.

In Table 3,** iNoun** and** iVerb** denote the sets of nouns and verbs related to interaction, respectively, which are extended from the original ones presented by Plake et al. Wildcard “*∗*” indicates any word or words in a pattern. The number of words that is substituted by a wildcard in a pattern is limited to five.

## 3. Method

### 3.1. Dominant Keywords

In a variety of the features mentioned in the preceding section, the feature* keyword* is used in most of the researches related to PPI extraction from the literature [9, 10]. This suggests that* keyword* provides strong evidence for the existence of PPIs. Although such a feature plays a very effective role in determining whether the sentence includes PPI, emphasizing only this feature might cause a side effect where other features are not utilized effectively. Therefore, in this paper, we make a distinction between two cases: the case when the* keyword* becomes a good index for classification and the case when the* keyword* does not become a good index for classification. We deal with these two cases by generating separate classifiers. We call the former* a dominant keyword*.

Note that the same keyword can be both dominant and not depending on its contexts. For example, in sentence “GerE binds to a site on one of these promoters, cotX, that overlaps its -35 region,” the value of feature* keyword* “bind” becomes important evidence for identifying the PPI between proteins GerE and cotX. Therefore, “bind” can be regarded as a dominant keyword in this case. However, in sentence “Neurocalcin a member of this family is an N-myristoylated calcium-binding protein that directly interacts with actin in a calcium-dependent manner,” the keyword “bind” is not related directly to the PPI between proteins Neurocalcin and actin. In this case, “bind” is not regarded as a dominant keyword, but instead keyword “interact” is dominant.

### 3.2. Keyword Position

As described in Section 3.1, the feature* keyword* shows an important role in generating classifiers that determine the presence or absence of PPI. Not only the content of the* keyword*, but also the structure of a sentence where it occurs is important. We can grasp the rough structure of a sentence by the feature* position of the keyword* showing the word order of a pair of protein names and the keyword constituting the instance. If the rough structures of the sentences differ, the features that should be emphasized in the process of generating classifiers will differ. Therefore, like dominant keywords, generation of separate classifiers becomes effective.

For example, if the value of feature* position of the keyword* is “infix,” the keyword exists between a pair of protein names in the sentence. In this case, a typical S-V-O sentence structure is often observed in which the protein names correspond to a subject and an object and the keyword corresponds to a verb. On the other hand, if the value of feature* position of the keyword* is “prefix” or “postfix,” the sentence is considered to have an atypical sentence structure, such as an inverted structure, the parallel expression of protein names, and phrase expression. In a typical sentence structure, since the relation of a protein name and the keyword is that of subject and verb or object and verb, such a feature as the* word distance* that provides the distance between the keyword and protein names plays an important role. However, in an atypical sentence structure, since such correspondence is not satisfied, the feature* word distance* is not always emphasized. Thus, depending on the sentence structure, the importance of the feature may change.

### 3.3. PPI Prediction Based on the Division of Training Set

As mentioned in Sections 3.1 and 3.2, depending on whether the sentence of the instance includes a* dominant keyword* and the word order of the keyword and protein names (i.e., the* position of the keyword*) in the sentence of the instance, the feature that should be emphasized will change. For this reason, we divide the training set into four categories, II, IP, NI, and NP, in Table 4 based on the existence of the* dominant keyword* and the* position of the keyword*. As a result, four classifiers are generated from each corresponding training subset divided from the original training set.

**Figure 2:**
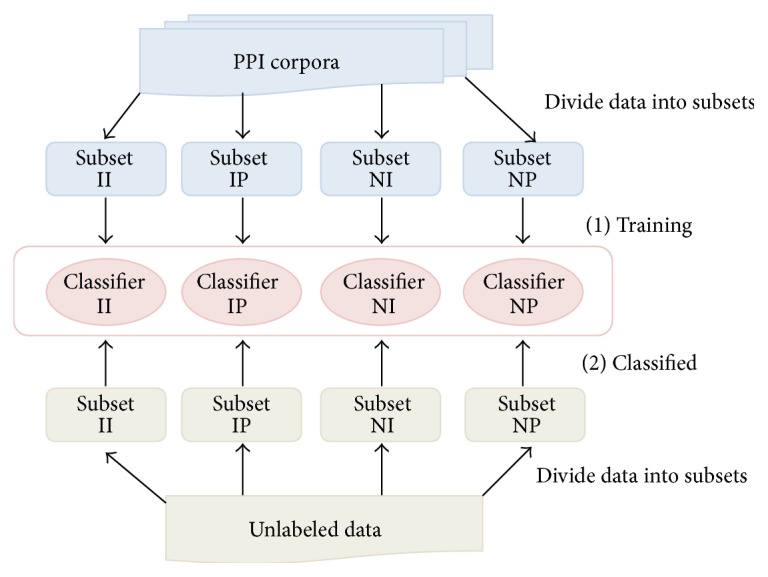
Overview of PPI prediction based on division of training set.

We also classify the unlabeled instances that need to determine the presence or the absence of a PPI into one of four categories and use the corresponding classifier to predict PPI. An overview of this process is shown in Figure 2.

### 3.4. Method of Dividing Training Set

#### 3.4.1. Overview

The division of a training set based on the appearance order of the protein names and the keyword in the sentence can be performed straightforwardly by referring to the feature* position of the keyword* of each instance. As mentioned above, however, a keyword is dominant in several instances but the same keyword is not necessarily dominant in other instances. Therefore, the division of a training set based on dominant keywords is not simple. We predict whether a dominant keyword is included in each instance and divide the training set based on the results of this prediction.

Next we outline our method that predicts the presence or absence of dominant keywords. By utilizing the presence of a keyword that easily becomes dominant and is less likely to become dominant, we assume that it is a dominant keyword in each instance. At the first stage of the assumption, each keyword is tentatively determined to be a dominant keyword or not to be a dominant keyword. Next, the training set is divided into two subsets: instances assumed to possess dominant keywords and instances that are not assumed to possess dominant keywords. These two subsets are used to generate two different classifiers. Then, by evaluating the success and failure of the classification results from these two classifiers, we verify whether the assumption of the presence or absence of the dominant keywords is appropriate and update this assumption. We later discuss the details of this update process. By repeating the assumption and verification processes, we improve the accuracy of predicting the existence or the nonexistence of dominant keywords for each instance.

#### 3.4.2. Generation of Assumption about Presence or Absence of Dominant Keywords

The initial assumption about whether a certain keyword is dominant is given by the observation of the bias of the classes of instances when classifying them based on the presence or absence of this keyword. We define unbalance degree *U*(*K*) for the value of feature* keyword K* by the following formula:

(1) $$\begin{array}{c} U\left(\;K\;\right)=\frac{\text{\# of positive instances containing }K}{\text{\# of all instances containing }K}. \end{array}$$

For certain keyword *K*, *U*(*K*) = 0.5 means that *K* is completely balanced. Conversely, *U*(*K*) = 0 or *U*(*K*) = 1 means that *K* is completely unbalanced. Therefore, when the value of *U*(*K*) or 1 − *U*(*K*) is less than predefined threshold *T*, *K* is initially regarded as a dominant keyword. We assume that the instance containing *K* possesses the dominant keyword; the instance that does not contain *K* does not possess a dominant keyword. In the following, we assign to each instance the *DK* value: *DK* = 1 for an instance assumed to possess a dominant keyword and *DK* = 0 for an instance that is assumed to not possess a dominant keyword.

#### 3.4.3. Verification and Updates of *DK* Values

**Figure 3:**
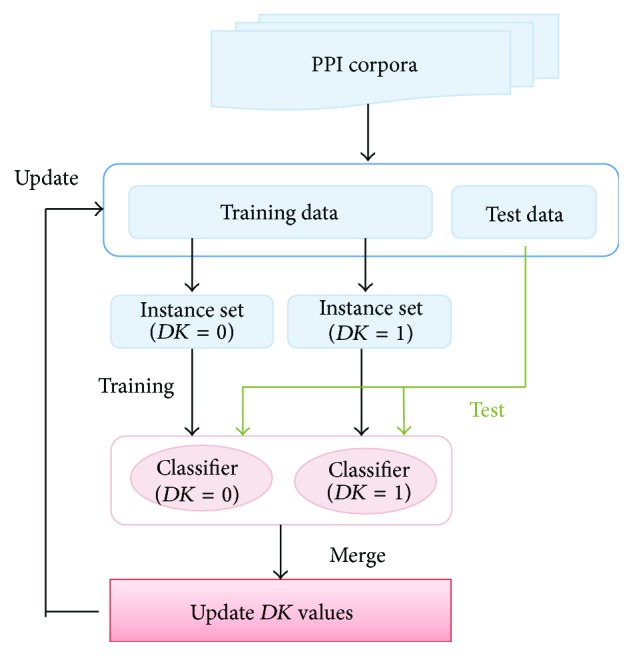
General flow of updating *DK* values.

The training set is divided into two subsets based on the *DK* values. Then two classifiers, *C*_0_ and *C*_1_, are generated from a subset containing only instances where *DK* = 0 and a subset of instances where *DK* = 1. Ideally, the former becomes a classifier that emphasizes the contribution of the dominant keyword, and the latter is a classifier with little consideration for its influence. Using these two classifiers, we classify the instances that are not used in training (test data). Based on the classification result from these two classifiers, we verify the assumption about the *DK* value of each instance, that is, the assumption about whether it contains the dominant keyword, and update this assumption. Repeating this process can improve the prediction accuracy of the *DK* value. An outline of the update method for *DK* values is shown in Figure 3.

To update *DK* values using these two classifiers, we utilize the framework of *k*-folds Cross Validation (CV). The pseudocode of the update algorithm is shown in Algorithm 1. **W** is a set of instances for updating *DK* values. The procedure **W**.splitCV(*j*) represents an operation that returns *j*th subset **w**_*j*_ when dividing **W** by *k*-folds CV. The procedure **w**_*j*_.update*DK*(**T**) represents an operation that updates the *DK* value of each instance in **w**_*j*_ by two classifiers, *C*_0_ and *C*_1_, generated from training set **T** (as described above, classifier *C*_0_ is generated by training only the instances possessing *DK* = 0 from **T**, and classifier *C*_1_ is generated by training only the instances possessing *DK* = 1 from **T**). In addition, preset *m* represents the number of iterations to perform CV.

The details of procedure** w**.update*DK*(**T**) that updates the *DK* value of each instance in **w** based on classifiers *C*_0_ and *C*_1_ generated from training set **T** are shown as follows.

For every instance in **w**, we predict whether that instance is positive or negative by classifiers *C*_0_ and *C*_1_. Since the correct answer for every instance of **w** is known beforehand, we can confirm whether this prediction is correct. There are three possible cases as follows:

- The prediction results of the two classifiers are different.
- Both of the prediction results are correct.
- Both of the prediction results are incorrect.

**Case 1 (the prediction results of the two classifiers are different).**

The fact that the prediction results of the two classifiers are different means that the impact of the presence or the absence of the dominant keyword on the classifiers is high. Therefore, we can determine that if a certain instance is predicted accurately by classifier *C*_1_ but inaccurately by classifier *C*_0_, the keyword of this instance is more likely to be dominant. Hence, regardless of its current *DK* value, we update its *DK* value to 1. On the contrary, if a certain instance is predicted accurately by classifier *C*_0_ but inaccurately by classifier *C*_1_, this instance shows a tendency similar to the instance that possesses no dominant keyword, and we update the *DK* value of this instance to 0 despite its current *DK* value.

**Case 2 (both of the prediction results of the two classifiers are correct).**

If both of the prediction results of the two classifiers are correct, we can only predict the class of the instance from features other than* keyword* regardless of the impact of the presence or the absence of the dominant keyword on the classifiers.

On the other hand, after a certain number of iterations have updated the *DK* value, the *DK* value often does not change. This means that the *DK* value converges to the correct value, or it is not correct and might have fallen in a local stable state. Therefore, the instance predicted correctly by both classifiers can escape the local state by randomly changing the *DK* values. In other words, we reverse the *DK* value by fixed probability *α* that we call the mutation rate for a negative instance that belongs to the actual negative class (True Negative). The reason why we are concerned with only the negative instances is simply that the number of positive instances is originally small. In such a situation, assigning *DK* values with incorrect randomness greatly decreases the extraction accuracy.

**Case 3 (both of the prediction results of the two classifiers are incorrect).**

If the prediction results of the two classifiers are both incorrect, the instance does not possess any valid dominant keyword, or the prediction from the features besides* keyword* is also hard. Therefore, removing such instances improves the overall prediction accuracy. However, the instances to be removed are limited to those that belong to the negative class (i.e., False Positive). The reason why we are concerned with only the negative instances is the same as above. Removing instances from the small positive set influences the extraction accuracy negatively.

### 3.5. Prediction of *DK* Values for Unlabeled Instances

As explained in Section 3.3, the original training set is divided into four categories using the updated *DK* values and the word order to generate four classifiers. To extract PPI, we must decide which of these four classifiers to apply to predict the class label of each unlabeled instance. However, unlike the training set, it is not possible to determine the *DK* values of the unlabeled instances in advance. Therefore, we also predict the *DK* values of the unlabeled instances. In other words, based on the training set possessing the updated *DK* values, we generate a new classifier, named *DK*-classifier, by considering the *DK* value of each training instance as its class label. Finally the *DK* values of the unlabeled instances are decided using the generated *DK*-classifier.

### 3.6. Applying Feature Selection to Training Set

In the above framework, because the original training set is divided into four training subsets, not all of the features are always valid for each training subset. Therefore, we only consider the selection of the valid features from all of the prepared features for each training subset. Generally, such a process is called feature selection, and various methods for it have been proposed. Instead of applying such methods, however, we adopt a simple manual method of feature selection in which meaningless or redundant features are eliminated beforehand based on the sentence structure by focusing on the division of the original training set based on the presence or the absence of the dominant keywords and word order. Lists of the features removed for each category of the training subsets are shown in Table 5.

As shown in Table 5, all of the removed features are eliminated based on the word order that represents the positional relation between the keyword and the protein pair. Since the value of feature* position of the keyword* is infix in subsets II and NI, typical S-V-O sentence structures in which the protein names correspond to a subject and an object and the keyword corresponds to a verb are often observed. Patterns 7, 8, 9, and 13 do not fit this S-V-O sentence structure. Patterns 7, 8, and 9 represent phrases in which the protein pair (*P*1, *P*2) and the keyword (*K*) are aligned by *K*-*P*1-*P*2. Moreover, pattern 13 becomes redundant, and its value is always 0. Similarly, since the value of feature* position of the keyword* is prefix or postfix in subsets IP and NP, such atypical sentence structures as inverted structure, parallel expression of protein names, and phrase expressions are often observed. In other words, patterns 1, 2, 10, and 12 do not fit these sentence structures. Therefore, we remove such features in advance.

## 4. Experimental Results

### 4.1. Evaluation Methods

We use five PPI corpora, LLL [13], HPRD50 [11], IEPA [14], AImed [1], and BioInfer [15], which are often used for PPI extraction evaluations. We evaluate five methods to confirm the effectiveness of our method using division into subsets, the prediction of the presence or the absence of the dominant keyword in every instance, and the process of feature selection proposed in previous sections. We used the Random Forest algorithm for generating classifiers whose extraction performance is relatively high and whose execution speed is also good.

Furthermore, let threshold value *T* be given by *T* = 0.15 for initially assuming the presence or the absence of dominant keywords (Section 3.4.2). If the value of threshold *T* is too low, very few numbers of instances with *DK* = 1 remain in the training set. As a result, a training set that is labeled either “negative” or “positive” may be empty or too small to build classifiers. On the other hand, if the value of *T* is too high, not only imbalanced keywords but also balanced ones are considered initial dominant keywords, which may lead to the deterioration of classification accuracy. Therefore, we searched for the smallest value of *T* that can generate classifiers for all of the PPI corpora used in the experiment and selected *T* = 0.15. The influence of the value of *T* on PPI extraction accuracy is discussed below.

Let the number of folds, *k*, the number of iterations, *m*, and mutation rate, *α*, of the Cross Validation in the procedure for updating the dominant keyword values (Section 3.4.3) be given by *k* = 10, *m* = 5, and *α* = 5%.

*(i) Single Classifier Method (SC)*. A single classifier is learned using the whole training set. PPI information is extracted by this classifier. 

*(ii) Multiple Classifiers Method (MC)*. The training set and the unlabeled instances are divided into four subsets by the criteria shown in Table 4, provided that the existence of the dominant keyword is determined simply by the unbalance degree for it. Then four classifiers are generated separately from these subsets. 

*(iii) Dominant Keyword-Based MC Method (DK-MC)*. The existence of the dominant keyword in every instance is predicted (Section 3.4). Based on the prediction, the training set and the unlabeled instances are divided into four subsets from which four classifiers are generated. 

*(iv) Feature Selection-Based MC Method (FS-MC)*. Feature selection is applied after dividing the training set and the unlabeled instances into subsets in the MC method. 

*(v) Dominant Keyword and Feature Selection-Based MC Method (DK-FS-MC)*. Feature selection is applied after dividing the training set and the unlabeled instances into subsets in the DK-MC method. 

We used evaluation data created from the above five corpora and divided them into training and test datasets to apply 10-fold CV. To evaluate the test data, we also used average Recall, Precision, and *F*-value defined by the following formulas:

(2) Recall=TPTP+FN,Precision=TPTP+FP,F-value=2∗Recall∗PrecisionRecall+Precision,

where TP, which denotes True Positive, is the number of correctly predicted instances of interacting protein pairs. FP and FN, which denote False Positive and False Negative, are the number of incorrectly predicted instances of noninteracting protein pairs and interacting protein pairs, respectively.

### 4.2. Evaluation Results

Table 6 shows the experimental results of all the methods mentioned in Section 4.1. Bold letters show the top score in each method.

*(i) SC and MC*. Comparing the extraction accuracy of* MC* and* SC*, even though some improvements in the *F*-values are observed in three corpora (LLL, IEPA, and AImed), the *F*-values remain unchanged or slightly decrease in the two remaining corpora.

Although* MC* performs learning by dividing the training set and the unlabeled instances into subsets, we can see that only determining the presence or the absence of the dominant keywords based on the bias of the occurrence of the keywords leads to inaccurate division. 

*(ii) DK-MC*. Comparing the extraction accuracy of the* DK-MC* and* MC* methods, the *F*-values increase in four corpora, LLL, HPRD50, IEPA, and BioInfer, but decrease slightly in AImed. However, the *F*-values are improved when comparing* DK-MC* with* SC*. This means that* DK-MC* is effective on the whole.

Predicting the presence or the absence of the dominant keyword in each instance can improve the learning performance compared with uniformly setting up the presence or the absence of the dominant keyword in each feature value. 

*(iii) FS-MC*. Comparing the extraction accuracy of the* FS-MC* and* MC* methods, the *F*-values increased in four corpora: LLL, HPRD50, IEPA, and BioInfer. Although the *F*-values decreased in AImed, they improved when comparing* FS-SC* and* SC* methods. This means that the performance of the feature selection acts effectively. Since more effective subsets of the features are selected for each subset divided from the training set by feature selection, the learning accuracy of each classifier is improved. However, since the improvement rate of the *F*-values in the AImed corpus declined significantly compared with the* DK-MC* method, we infer that dominant keywords exert a much greater influence on the AImed corpus than the performance of the feature selection. 

*(iv) DK-FS-MC*. Our* DK-FS-MC* method, which incorporates all of the contents proposed above, integrates the idea of the* DK-MC* and* FS-MC* methods. Comparing the* DK-MC* and* FS-MC* methods, *F*-values are improved considerably in HPRD50 and BioInfer or improved slightly or equivalently in the remaining corpora.

In the AImed corpus, although the* MC* method shows the top score of *F*-values, the difference between the *F*-values of the* DK-FS-MC* and* MC* methods is only 0.3 percentage points, and the *F*-value of the* DK-FS-MC* method is adequately improved compared with the SC method. In the LLL, HPRD50, IEPA, and BioInfer corpora, the* DK-FS-MC* method outperforms the other methods based on *F*-values. Consequently, the prediction of the presence or the absence of dominant keywords and the performance of the feature selection for each subset simultaneously very effectively improved the extraction accuracy. 

We explored the influence of the value of threshold *T* on PPI extraction accuracy in our proposed method.  Table 7 shows the *F*-values of the* DK-FS-MC* method when changing the value of *T* from 0.15 to 0.35. Although there is a slight difference in degree depending on the corpus, on the whole the lower the value of *T* is, the higher the extraction accuracy is. This result shows that *T* = 0.15 is appropriate.

### 4.3. Comparison with Existing Methods

The comparison results of our proposed method (*DK-FS-MC* method) with existing works on the extraction of PPI information are shown in Table 8. The* DK-FS-MC* method shows the best *F*-values in LLL and HPRD50 and relatively comparable results in IEPA and AImed. The *F*-value of the* DK-FS-MC* method surpasses the related works in Table 8 from 3% to 7% in LLL and surpasses the method proposed by Van Landeghem et al. [6] by 6% in HPRD50.

Fundel et al. [11] simply employed three rules to extract paths connecting two entities from dependency parse trees. They did not use as many kinds of features as we did, for example, lexical features, features from constituent parse trees, and dominant keywords. Similarly, Fayruzov et al. [5] did not utilize any lexical features, including specific interaction keywords and especially dominant keywords as well as features that use existing patterns. They proposed a method based solely on complete parsing information derived from both dependency parse trees and constituent parse trees. Therefore, the *F*-values of our method are far better than the Fundel et al. and Fayruzov et al. methods in both LLL and AImed.

Although the *F*-values of the* DK-FS-MC* method are better than the method by Van Landeghem et al. in LLL and HPRD50, the results of their method outperformed ours in IEPA and AImed from 1.8% to 2%. They utilized rich feature vectors derived from dependency graphs and applied feature selection. However, their feature sets are somewhat huge. The numbers of features of their method in LLL, HPRD50, IEPA, and AImed are 1,600, 2,600, 6,900, and 14,000, respectively. Even when they applied feature selection, the number of features is at least 200. Therefore, their method resulted in high-dimensional sparse feature vectors. Moreover, the number of features of their method is not equal across all corpora and seems subject to the size of each corpus. The number of features of our method in LLL, HPRD50, IEPA, and AImed was only 44 and is equal across all corpora. Since the number of features they applied across all the corpora is too big, the time necessary to build the classifier becomes too long. They reported that the time required to build a support vector machine classifier, excluding the time for feature extraction, is from 3 hours 22 minutes to 6 hours 5 minutes in AImed, whereas the time to build the classifier in our method is only about one minute. This shows that, in general, our method is better than theirs for balancing extraction accuracy and execution time.

It is not easy to compare the results in AImed with other related research due to different preprocessing and feature extraction ways. For example, although the method by Giuliano et al. utilized neither features obtained from parsing information, nor features using existing patterns, nor dominant keywords, their *F*-value is higher than ours in AImed. They designed a combination of kernels: (1) a global context kernel that uses the information, which is related to tokens before, between, and after the two proteins and is represented by a bag-of-words; (2) a local context kernel that considers the order of the tokens [2]. In their method, however, the protein names are partly visible. This influences the learning performance unlike our method in which the protein names are always blind. This difference makes the *F*-value of their method higher than ours in AImed.

Mitsumori et al. [3] used three bag-of-word features related to the following: (1) left-side words; (2) right-side words; and (3) middle words of two protein names. They showed that three words to the left-side (and right-side) is the optimal number of tokens in context, which yields the best *F*-score. Giuliano et al. and Mitsumori et al. only counted the multiple occurrences of the same interaction pair in a document one time. Conversely, the correct interaction must be extracted for each occurrence of the same interaction pair in a document in our method. As a result, the method by Giuliano et al. produced higher *F*-value performance than ours in AImed.

Erkan et al. [12] represented instances by dependency parse trees. They measured the similarity of two instances by two distinct kernel functions based on cosine similarity and edit distance, which are calculated on the two paths between the protein names of the two instances.  Table 9 shows the number of positive and negative pairs in the AImed corpus. Erkan et al., Giuliano et al., Mitsumori et al., Fayruzov et al., and Van Landeghem et al. applied fewer negative pairs than us in the AImed corpus. Giuliano et al. and Mitsumori et al. applied more positive pairs than us in the AImed corpus. This is one reason that boosts the performance of the *F*-values of the methods by Giuliano et al. and Van Landeghem et al., compared with the* DK-FS-MC* method. Despite these great differences, by utilizing dominant keywords, our* DK-FS-MC* method still outperforms the edit of Erkan et al., the cosine of Erkan et al., Fayruzov et al., and Mitsumori et al. methods based on the *F*-values in AImed.

## 5. Conclusion

In this paper we described our automatic extraction method for PPI from scientific articles based on dominant keywords that considerably contribute to learning and classification. Based on the existence of dominant keyword and sentence structure, a training set is divided into four subsets and four classifiers are generated from each training subset.

We introduce a mechanism that can predict whether the mentioned keyword is dominant for each instance. Initially, a particular keyword is assumed to be dominant based on the bias of the classes. Then two classifiers are generated by training the two subsets divided from the training set. Based on the classification result, the assumption of the existence of a dominant keyword that was assumed previously is verified and updated. By repeating this process, we implemented more accurate predictions about dominant keywords. Moreover, we performed feature selection in which redundant features are removed beforehand based on the sentence structure to improve the extraction accuracy.

Through experimental results, we showed that dominant keyword prediction greatly improves the accuracy of PPI extraction. Moreover, the DK-FS-MC method shows good results of *F*-values in two corpora compared with related methods that did not introduce neither dividing the training set into subsets, predicting the existence of dominant keywords in every instance, nor the feature selection process.

Extraction accuracy is influenced by the unbalance of PPI data. Since we used the Random Forest algorithm, we cannot tackle this challenge of unbalanced PPI data. In ongoing work, we will apply the Weighted Random Forest or the Balanced Random Forest [16] to unbalanced PPI data to improve the PPI extraction accuracy.

## Figures and Tables

**Algorithm 1:**
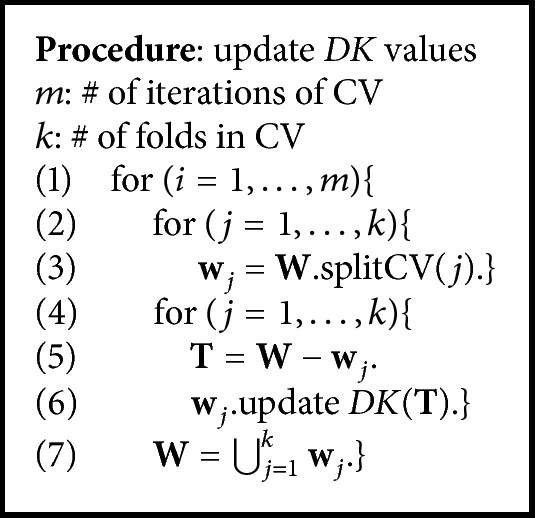
Procedure for updating *DK* values.

**Table 1:** Features obtained directly from sentences.

Features	Definitions/remarks	Values	Examples
Keywords	Words representing relationship between two proteins	One of the 180 kinds of words obtained by stemming 642 kinds of words such as * “interact”*, * “bind”*, * “active”*, and * “depend”*, observed frequently in sentences describing PPI	

Distance between protein pair and keyword: three types	The word distance defined by the number of words appearing between keyword mentioned above and protein names constituting the protein pair; Type 1 is the distance between *P*1 and *K*, Type 2 is the distance between *P*2 and *K*, and Type 3 is the distance between *P*1 and *P*2	Integer value	In sentence * “P1 is driven by P2”*, if “driven” is the keyword, Type 1, Type 2, and Type 3 are 1, 1, and 3, respectively

Position of keyword: three types	The word order of protein pair and keyword	“Infix” (the order of the sentence is [*P*1-*K*-*P*2]), “prefix” (the order of the sentence is [*K*-*P*1-*P*2]), or “postfix” (the order of the sentence is [*P*1-*P*2-*K*])	In sentence * “P1 is driven by P2”*, the feature value is “infix”

Position of protein names	The value adding word distance between the word at the beginning of the sentence and the protein name to one; positions 1 and 2 are defined for *P*1 and *P*2	Integer value	In sentence * “P1 is driven by P2”*, Position 1 is 1 and Position 2 is 5

Comma between keyword and protein pair: four types	Since the topic often changes before and after a comma, we use such information if there is any comma between the keyword and the protein pair	“yy”, “nn”, “yn”, or “ny” (e.g., “yy” means commas are observed between *A* and *B* and between *B* and *C*, where *A*, *B*, and *C* denote three words: the keyword and two protein names constituting the protein pair)	In sentence * “P1 is driven by P2”*, the feature value is “nn”

Negative words	Whether any negative word such as * “not”*, * “unable”*, or * “incapable”* appears between the protein names, or the keyword and the protein name	“True” or “false”	In sentence * “P1 is not driven by P2”*, the feature value is “true”

Conjunctive words	Whether one of the following 16 kinds of words representing conjunctive relations appears: * “where”*, * “when”*, * “what”*, * “why”*, * “how”*, * “as”*, * “though”*, * “although”*, * “because”*, * “so”*, * “therefore”*, * “hence”*, * “since”*, * “wherein”*, * “whereas”*, and * “whereby”*	“True” or “false”	In sentence * “P1 is not driven by P2”*, the feature value is “false”

“Which”	Whether “which” appears; since “which” also represents the conjunctive relation but occurs more frequently than the 16 words mentioned above, we distinguish “which” from the above features	“True” or “false”	

“But”	Whether “but” appears; in addition to “which”, “but” also frequently represents the conjunctive relation; however, “but” introduces negation to the context	“True” or “false”	

Words representing assumptions or conditions	Whether “if” or “whether” appears between the protein names or the keyword and the protein name	“True” or “false”	

Preposition of keyword	The preposition following the keyword providing that the word distance between the keyword and the preposition is within 3; if there are many prepositions, the preposition is used whose word distance from the keyword is nearer	One of the prepositions	In sentence “*P1 is driven by P2*”, the feature value is “by”

Multiple occurrences of keywords	Whether there is more than one keyword in a sentence	“true” or “false”	In sentence “*P1 is driven by P2*”, the feature value is “false”

Second keywords: seven kinds	Only one of seven particular words: “*bind*”, “*interact*”, “*regulate*”, “*induce*”, “*stimulate*”, “*associate*”, and “*known*” is not selected as a keyword, whether that word appears between the protein names; compared with other keywords, these seven words can be regarded as particularly important in PPI information and this feature prevents them from being overlooked as keywords	“True” or “false” for each of the seven words (if some of these seven words appear in the sentence and are not selected as a keyword, we use “true” as a feature value for them)	In sentence “*P1 binds P2*”, since “bind” is already selected as a keyword, the feature value of the second keyword (bind) is “false”; since no other words are included in the sentence, the feature value of each is also “false”

Parallel expression of protein pair	Whether the protein names constituting the protein pair are adjacent (they are also considered adjacent even if “—”, “/”, “and”, “or”, “(” appears between them); if protein names are expressed in parallel in a sentence, interaction between them is difficult; we can easily determine the parallel expression of a protein pair in a sentence by determining whether these protein names are adjacent in the word order of that sentence	“True” or “false”	In sentence “*Protein binds P1 or P2*”, the feature value is “true”

**Table 2:** Features obtained from parsing information.

Features	Definitions/remarks	Values	Examples
Height of protein pair and keyword: three types	The heights of the protein names constituting the protein pair and the keyword at the parse tree structure: these heights differ from word distances; features height_*P*1, height_*P*2, and height_*K* are defined for the heights of *P*1, *P*2, and *K*, respectively	Integer value	In Figure 1, height_*P*1, height_*P*2, and height_*K* are 2, 5, and 3, respectively

Part-of-speech information of protein pair and keyword: three types	The part-of-speech information of PATH (the path from the root) at the parse tree structure of the protein names constituting the protein pair and the keyword; it is possible to represent the syntax structure and train classifiers to learn pseudo grammar structure; features POS_*P*1, POS_*P*2, and POS_*K* are defined for the part-of-speech information of the PATH of the leaf representing *P*1, *P*2, and *K*, respectively	List of part-of-speech information of PATH	In Figure 1, POS_*P*1, POS_*P*2, and POS_*K* are “NP, NN,” “VP, VP, PP, NP, NN,” and “VP, VP, VBN,” respectively

**Table 3:** Set of 13 PPI patterns.

Number	PPI pattern
Pattern 1	** P1** *∗* **iVerb** *∗* **P2**
Pattern 2	** P1** *∗* **iVerb** *∗* by *∗* **P2**
Pattern 3	** iVerb** of *∗* **P1** *∗* by *∗* **P2**
Pattern 4	** iVerb** of *∗* **P1** *∗* to *∗* **P2**
Pattern 5	** iNoun** of *∗* **P1** *∗* [by∣through] *∗* **P2**
Pattern 6	** iNoun** of *∗* **P1** *∗* [with∣to∣on] *∗* **P2**
Pattern 7	** iNoun** between *∗* **P1** *∗* and *∗* **P2**
Pattern 8	complex between *∗* **P1** *∗* and *∗* **P2**
Pattern 9	complex of *∗* **P1** *∗* and *∗* **P2**
Pattern 10	** P1** *∗* form *∗* complex with *∗* **iVerb** *∗* **P2**
Pattern 11	** P1** *∗* **P2** *∗* **iNoun**
Pattern 12	** P1** depend of ** P2**
Pattern 13	between ** P1** and ** P2**

**Table 4:** Division of training set.

Subset	Dominant keyword	Position of keyword
II	Included	Infix
IP	Included	Prefix/postfix
NI	Not included	Infix
NP	Not included	Prefix/postfix

**Table 5:** Removed features for each training subset.

Subset	Removed features
II	Patterns 7, 8, 9, and 13
IP	Patterns 1, 2, 10, and 12
NI	Patterns 7, 8, 9, and 13
NP	Patterns 1, 2, 10, and 12

**Table 6:** Experimental results.

Corpus	LLL			HPRD50			IEPA			AImed			BioInfer		
(%)	*R*	*P*	*F*	*R*	*P*	*F*	*R*	*P*	*F*	*R*	*P*	*F*	*R*	*P*	*F*
SC	85.4	79.1	82.1	70.1	75.2	72.3	63.6	68.9	66.1	49.5	67.9	57.3	67.7	74.8	71.1
MC	85.4	81.9	83.6	72.4	71.5	72.0	64.2	70.3	67.1	54.4	67.7	**60.3**	68.1	74.3	71.1
DK-MC	84.8	84.8	84.8	77.3	72.8	75.0	66.9	71.3	69.0	54.4	66.8	60.0	69.5	74.3	71.7
FS-MC	87.8	81.8	84.7	77.3	73.7	75.4	65.6	72.6	69.0	51.8	66.7	58.3	69.1	75.0	71.9
DK-FS-MC	86.6	83.5	**85.0**	77.9	76.0	**77.0**	67.2	71.4	**69.2**	55.0	66.0	60.0	70.8	74.8	**72.7**

**Table 7:** Influence of value of *T* on *F*-values.

*T*	LLL	HPRD50	IEPA	AImed	BioInfer
0.15	** 85.0**	77.0	** 69.2**	** 60.0**	** 72.7**
0.20	83.7	** 77.6**	68.4	59.4	71.6
0.25	81.6	74.6	67.7	58.9	71.6
0.30	83.3	72.0	67.5	** 60.0**	71.9
0.35	84.3	73.0	66.1	59.3	71.9

**Table 8:** Performance comparison of PPI extraction.

Corpus	Method	*R*	*P*	*F*
LLL	Fundel et al. [11]	79.0	**85.0**	82.0
	Fayruzov et al. [5]	86.0	72.0	78.0
	Van Landeghem et al. [6]	84.0	79.0	82.0
	DK-FS-MC	**86.6**	83.5	**85.0**

HPRD50	Van Landeghem et al. [6]	71.0	71.0	71.0
	DK-FS-MC	**77.9**	**76.0**	**77.0**

IEPA	Van Landeghem et al. [6]	**69.0**	**74.0**	**71.0**
	DK-FS-MC	67.2	71.4	69.2

AImed	Giuliano et al. [2]	**63.2**	64.5	**63.9**
	Mitsumori et al. [3]	53.6	55.7	54.3
	Fayruzov et al. [5]	50.0	41.0	45.0
	Van Landeghem et al. [6]	58.0	66.0	62.0
	Edit of Erkan et al. [12]	43.5	**77.5**	55.6
	Cosine of Erkan et al. [12]	55.0	62.0	58.1
	DK-FS-MC	55.0	66.0	60.0

**Table 9:** Number of positive/negative pairs in AImed corpus applied in our work and existing works.

	Our work	Mitsumori et al. [3]	Giuliano et al. [2]	Van Landeghem et al. [6]	Erkan et al. [12]	Fayruzov et al. [5]
Positive pairs	1,000	1,107	1,008	1,000	951	816
Negative pairs	4,834	4,369	4,634	4,670	3,075	3,204
